# Final report on North Central Cancer Treatment Group N0877 (alliance): A phase II randomized, placebo-controlled trial of chemoradiotherapy with or without dasatinib for glioblastoma

**DOI:** 10.1093/neuonc/noaf156

**Published:** 2025-06-28

**Authors:** William G Breen, Jesse G Dixon, S Keith Anderson, Jann N Sarkaria, Paul D Brown, Elizabeth S Yan, Sani Kizilbash, Eva Galanis, Daniel Anderson, David Tran, Miroslaw Mazurczak, Derek R Johnson, Francois J Geoffroy, Clinton Leinweber, Nadia N Laack

**Affiliations:** Department of Radiation Oncology, Mayo Clinic, Rochester, MN, USA; Alliance Statistics and Data Management Center, Mayo Clinic, Rochester, MN, USA; Alliance Statistics and Data Management Center, Mayo Clinic, Rochester, MN, USA; Department of Medical Oncology, Mayo Clinic, Rochester, MN, USA; Department of Radiation Oncology, Mayo Clinic, Rochester, MN, USA; Department of Radiation Oncology, Mayo Clinic, Rochester, MN, USA; Department of Radiation Oncology, Mayo Clinic, Rochester, MN, USA; Department of Radiation Oncology, Mayo Clinic, Rochester, MN, USA; Office of the Alliance Group Chair, Mayo Clinic, Rochester, MN, USA; Regions Hospital, Metro Minnesota Community Oncology Research Consortium, St Louis Park, MN, USA; Division of Oncology, Washington University - Siteman Cancer Center, Saint Louis, MO, USA; Sanford NCI Community Oncology Research Program of the North Central Plains, Sioux Falls, SD, USA; Department of Radiation Oncology, Mayo Clinic, Rochester, MN, USA; Department of Medical Oncology, Mayo Clinic, Rochester, MN, USA; Illinois Oncology Research Associates, Peoria, IL, USA; East Carolina University, Greenville, NC, USA; Department of Radiation Oncology, Mayo Clinic, Rochester, MN, USA

**Keywords:** chemotherapy, clinical trial, glioblastoma, radiotherapy

## Abstract

**Background:**

Dasatinib is an oral inhibitor of the Src kinase family, with preclinical data indicating impact on gliomagenesis, tumor invasion, and radiosensitivity.

**Methods:**

North Central Cancer Treatment Group N0877 is a phase 1 dose escalation and phase II randomized study evaluating the maximum tolerated dose (MTD), safety, and efficacy of dasatinib with radiation and temozolomide (TMZ) for glioblastoma. Following identification of the MTD, adult patients with a histologic diagnosis of glioblastoma were randomized 2:1 between dasatinib given with standard concurrent and adjuvant TMZ, versus placebo with standard concurrent and adjuvant TMZ. Radiation dose was 60 Gy in 30 fractions. The primary endpoint was overall survival (OS). Secondary endpoints included progression-free survival (PFS), toxicity, and quality of life.

**Results:**

Thirteen patients were enrolled in the phase I component and established the MTD and phase II dose of 150 mg given daily. A total of 204 patients were enrolled in the phase II component. OS was not different between arms (median OS 15.6 months for dasatinib compared to 19.3 months for placebo, hazard ratio:1.21 favoring placebo, 95% CI: 0.88–1.65, log-rank *P*-value: .238). Similarly, PFS was not significantly different between the dasatinib and placebo arms. There was a significant increase in anemia, nausea, and creatinine elevation with dasatinib, but significantly more grade 3 lymphopenia with placebo.

**Conclusions:**

The addition of dasatinib to standard chemoradiation did not improve outcomes for patients with glioblastoma.

**Clinical Trial Identifier:**

NCT00869401.

**Key Points**

- Dasatinib with chemoradiation did not improve survival for glioblastoma.
- Dasatinib was relatively well tolerated by patients.
- Improved ability to identify agents most likely to succeed in patients is needed.

**Importance of the Study**

This study describes a large clinical trial performed at multiple institutions testing whether the addition of dasatinib to standard chemoradiation improved overall survival for patients with glioblastoma. Dasatinib was well-tolerated but did not improve overall or progression-free survival, and should not be added to standard of care treatments in practice. A better understanding of why dasatinib and other agents with promising preclinical data have not translated to improved survival for patients with glioblastoma is needed.

Glioblastoma (GBM) is the most common primary brain tumor in adults and continues to carry a poor prognosis, with a median survival of less than 2 years despite current multimodality treatment.^1,2^ The therapeutic resistance of GBM stems from multiple factors, including radiation resistance, hypoxia-associated angiogenesis, and high degrees of tumor invasion, all of which are modulated by the Src-family kinases (SFK).^3–5^ SFK inhibitors have shown promising preclinical efficacy, resulting in destruction of tumor vasculature and reduced invasive potential, thereby significantly enhancing antitumor efficacy compared to radiation alone.^3,6^ Dasatinib (BMS-354825) is a potent oral ATP-competitive multi-targeted kinase inhibitor of all SFKs that has demonstrated preclinical promise in the treatment of glioma. While small prior studies have evaluated the efficacy of dasatinib in different settings, including recurrent glioblastoma, no large, randomized studies had previously been completed for newly diagnosed glioblastoma.^7–9^

Herein, we present the results of North Central Cancer Treatment Group (NCCTG) N0877, a phase I/II randomized placebo-controlled clinical trial evaluating the role of dasatinib incorporated with standard chemoradiotherapy in the treatment of glioblastoma. NCCTG is now part of the Alliance for Clinical Trials in Oncology (Alliance). The goal of the phase I study was to establish the maximum tolerable dose (MTD) of dasatinib combined with radiation therapy (RT) and temozolomide (TMZ) in patients with glioblastoma. The goal of the phase II portion was to determine the efficacy of dasatinib in combination with RT and TMZ, followed by adjuvant TMZ in patients with newly diagnosed glioblastoma, in comparison with the standard-of-care approach consisting of RT and TMZ followed by adjuvant TMZ.

## Methods

Each participant signed an IRB-approved, protocol-specific informed consent document in accordance with federal and institutional guidelines. Data collection and statistical analyses were conducted by the Alliance Statistics and Data Management Center. Data quality was ensured by review of data by the Alliance Statistics and Data Management Center and by the study chairperson, following Alliance policies. All analyses were based on the study database frozen on November 27th, 2019.

### Phase I

#### Study population.—

Adults (age ≥ 18 years) with newly diagnosed histologic glioblastoma (WHO 2007 classification) with Eastern Cooperative Oncology Group score of 0–2 were enrolled on a prospective dose-escalation study to determine the maximum tolerated dose (MTD) of dasatinib. Please see the protocol (Supplementary Material) for a complete list of inclusion and exclusion criteria. Of note, given the era in which the study was written and patients were enrolled, the current diagnostic criteria for glioblastoma, including isocitrate dehydrogenase (IDH) mutational status, were not utilized. This study was approved by the institutional review boards at all participating sites. Safety was monitored by the Mayo Clinic Cancer Center Data and Safety Monitoring Board, in conjunction with NCCTG, and later, the production of the annual Alliance Study Summary Report.

#### Dasatinib dosage assessment.—

A 3 + 3 design (3 to 6 patients treated at each dose level) was used to assess acute toxicity of dasatinib in combination with concomitant RT/TMZ. Doses were not escalated in any individual patient. Dose escalation steps were: 50 mg twice daily; 100 mg once daily in the morning, and 150 mg once daily in the morning. Patients were observed for dose-limiting toxicity (DLT) during concomitant dasatinib for the purpose of defining the MTD of daily oral dasatinib given concomitantly with RT/TMZ. DLT included ≥grade 3 non-hematologic adverse events (AEs), ≥grade 4 hematologic AE or radiation dermatitis, or failure to administer >75% of dasatinib/TMZ or interruption of RT for more than 5 days due to AEs.

The MTD was defined as the highest safely tolerated dose level where at most one out of 6 patients experienced DLT, with the next higher dose having at least 2 out of a maximum of 6 patients experience DLT. A total of 6 patients treated at the MTD were required to identify common toxicities at the MTD.

Time-related variables, such as time until any treatment-related toxicity, were also recorded. However, due to the small sample size in the phase I dose-escalation trial, the generalizable analyses were not planned, and data were only analyzed to inform the dose choice for phase II.

### Phase II

#### Study population.—

The same eligibility criteria described for phase I were also used for phase II. The primary efficacy analysis for the phase II portion of this trial was conducted using the intent-to-treat (ITT) population of all registered patients. Patients were stratified according to age for the phase II portion (>70 years vs. ≤70 years). A sensitivity analysis, including only those patients who were randomized and received at least one cycle of study treatment, was defined as the Modified ITT population.

#### Randomization procedure.—

After eligibility was confirmed, the patient was registered, and the stratification factor was recorded (age ≤ 70 vs. age > 70), the patient was randomized to one of 2 treatment groups using the Pocock and Simon dynamic allocation procedure to balance the marginal distributions of the stratification factors between groups.^10^ Randomization was in a 1:2 ratio: Group 1 (standard therapy arm) consisted of placebo combined with concomitant RT and TMZ, followed by adjuvant TMZ; Group 2 (experimental arm) consisted of dasatinib combined with concomitant RT and TMZ and adjuvant TMZ.

#### Treatment schedule.—

Supplementary Table 1 shows the phase II treatment schedule for both groups. Complete treatment details can be found in the protocol (Supplementary Material).

Cycle 1 consisted of concomitant RT and TMZ, plus either placebo (Group 1) or dasatinib (Group 2) taken once daily, beginning on the same day as RT and continuing for a total of 42 days. Radiotherapy was delivered using either intensity-modulated RT or 3-D conformal RT. A target volume including the tumor cavity and residual enhancement with a 1-cm clinical tumor volume expansion and a 3- to 6-mm planning target volume expansion was treated to 60 Gy in 30 fractions, while a target volume also including T2 hyperintensity with a 1-cm clinical tumor volume expansion and a 3- to 6-mm planning target volume expansion was treated to either 54 Gy in 30 fractions or 50 Gy in 25 fractions.

Cycle 2 was a 28–42 day rest period. Cycles 3–8 consisted of adjuvant TMZ, plus either placebo taken once daily or dasatinib taken once daily at the same dose level as the end of Cycle 1, and continuing for a total of 28 days per cycle. All subsequent cycles requiring adjuvant chemotherapy consisted of either placebo or dasatinib alone, once daily at the same dose as the end of the previous cycle, for a total of 28 days.

#### Safety stopping rules and AE monitoring.—

The stopping rule stated that if 3 out of the first 20 patients, or if at any time after the first 20 patients are enrolled ≥15% of patients developed ≥grade 4 non-hematologic AEs at least possibly related to study treatment, the study team would review the data to determine whether to suspend accrual, modify the dosage, continue further AE monitoring, or close the trial. Common Terminology Criteria for AEs v3.0 was used to determine grading for these stopping rules.

#### Overall survival.—

The primary endpoint for the phase II study was to compare overall survival (OS) between patients on the standard of care arm versus the experimental arm. OS was defined as the time from registration until death due to any cause, and was estimated using the Kaplan–Meier method and compared between treatment groups using the log-rank test. If the *P*-value of the log-rank test was shown to be greater than *P* = .1, the conclusion would be that RT/TMZ/dasatinib offers no benefit relative to standard RT/TMZ.

#### Progression-free survival and time-to-progression.—

Progression-free survival (PFS) was defined as the time from study registration to the date of the first observation of disease progression or death due to any cause, whichever came first. If a patient had not progressed or died, PFS was censored at the time of last follow-up. PFS rates by treatment arm were calculated at specific timepoints (eg, 12, 15, and 18 months) using the Kaplan–Meier method.

Time-to-disease progression (TTP) was defined as the time from the start of study therapy to documentation of disease progression. Patients who died without documentation of progression were considered to have had tumor progression at the time of death, unless there was documented evidence confirming no progression occurred before death. The TTP distribution was estimated using the Kaplan–Meier method.

#### AEs.—

The maximum Common Terminology Criteria for AEs version 3.0 grade for each type of treatment-related AE was recorded for each patient, and frequency tables for each arm were analyzed. All patients who received any study treatment were evaluated for AEs and treatment-related AEs and were included in the efficacy analyses. Kruskal–Wallis tests were used to compare the 2 arms.

#### Patient-reported quality of life.—

Quality of life (QOL) was assessed with 2 questionnaires: (1) FACT-Br and EORTC QLQ-C15-PAL, and (2) EORTC QLQ-BN20. Patients completed both questionnaires at baseline and at each MRI evaluation for a maximum of 6 evaluations (Cycles 4, 6, 8, 10, and 12). These timepoints were selected to correlate findings with radiologic and clinical evaluations.

QOL was computed using the corresponding scoring algorithms for each questionnaire at each collection time. Generalized linear mixed models that incorporated main effects of treatment arm, time and interaction of arm and time were applied to analyze the longitudinal data of QOL. In order to account for baseline confounding factors, these models were adjusted for age, sex, and corticosteroid therapy. Population-level and subject-level longitudinal plots were created to display the trend of patient-reported QOL; interaction plots were created to display the effects of treatment arm and time on QOL. An effect size of 0.5 was used to reflect changes in score in the FACT-Br and EORTC QLQ forms, which were deemed clinically meaningful.

## Results

### Phase I Study

#### Patient characteristics.—

From June 5th, 2009, through April 29th, 2011, 13 patients were accrued to the phase I dose-escalation study at dose level 0 (*n* = 3, 50 mg twice daily), dose level 1 (*n* = 3, 100 mg each morning), and dose level 2 (*n* = 7, 150 mg each morning). All patients completed treatment, and one patient was alive and censored for OS with 103 months of follow-up. The average age of patients was 55 years (range = 21–76 years), 9 (69%) were male, and 11 (84%) had undergone either a gross total or subtotal resection prior to enrollment. Eight (61%) were on corticosteroids at the time of enrollment.

#### Dose-limiting toxicities.—

One patient experienced DLTs at dose level 0 (grade 4 leukopenia, neutropenia, and thrombocytopenia), no patients experienced DLTs at dose level 1, and one patient experienced a DLT at dose level 2 (grade 3 rash). Accordingly, dose level 2 (150 mg taken every morning) was determined to be the MTD and was assigned as the starting dose for the phase II study.

### Phase II Study

#### Patient characteristics.—

**Figure 1.**
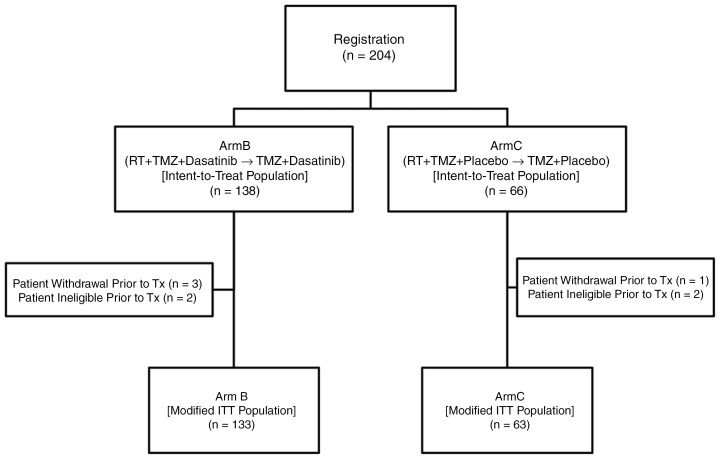
CONSORT diagram.

Phase II enrolled patients from August 31st, 2011, to January 14th, 2014. A total of 204 patients were consented to the trial, were randomized to a treatment arm, and were considered evaluable according to the ITT principle. Five patients in the experimental arm and 3 in the standard treatment arm dropped out before receiving any treatment, which resulted in a total of 196 patients included in modified ITT analyses (Figure 1, CONSORT Diagram). All registered patients were examined for baseline characteristics at the time of registration, and no significant differences were noted between arms (Table 1). Patients in the dasatinib arm received an average of 3.5 cycles of adjuvant temozolomide (standard deviation 2.15), and patients on the placebo arm received an average of 4.33 cycles (standard deviation 1.97).

**Table 1.** Patient Characteristics

	Dasatinib (*N* = 138)	Placebo (*N* = 66)	*P*-value
**Age**			.3396^1^
Mean (SD)	58.7 (11.29)	57.1 (10.89)	
Median (range)	60.0 (29.0, 82.0)	59.5 (29.0, 80.0)	
**Sex**			.5550^2^
Female	54 (39.1%)	23 (34.8%)	
Male	84 (60.9%)	43 (65.2%)	
**Race**			.6920^2^
White	130 (94.2%)	62 (93.9%)	
Black or African American	1 (0.7%)	1 (1.5%)	
Asian	3 (2.2%)	0 (0.0%)	
American Indian or Alaska Native	1 (0.7%)	0 (0.0%)	
Not reported	2 (1.4%)	2 (3.0%)	
Patient unsure	1 (0.7%)	1 (1.5%)	
**Ethnicity**			.3248^2^
Hispanic or Latino	7 (5.1%)	1 (1.5%)	
Not Hispanic or Latino	124 (89.9%)	64 (97.0%)	
Not reported	4 (2.9%)	1 (1.5%)	
Patient unsure	3 (2.2%)	0 (0.0%)	
**Performance status**			.9799^2^
0	50 (36.2%)	23 (34.8%)	
1	72 (52.2%)	35 (53.0%)	
2	16 (11.6%)	8 (12.1%)	
**Extent resection**			.7463^2^
Biopsy	21 (15.2%)	8 (12.1%)	
Subtotal resection	56 (40.6%)	30 (45.5%)	
Gross total resection	61 (44.2%)	28 (42.4%)	
**MGMT status**			.1930^2^
Unmethylated	22 (28.6%)	15 (45.5%)	
Methylated	42 (54.5%)	15 (45.5%)	
Indeterminate	13 (16.9%)	3 (9.1%)	
Missing	61	33	

^1^Kruskal–Wallis *P*-value^2^;Chi-Square *P*-value;.

#### Overall survival.—

**Figure 2.**
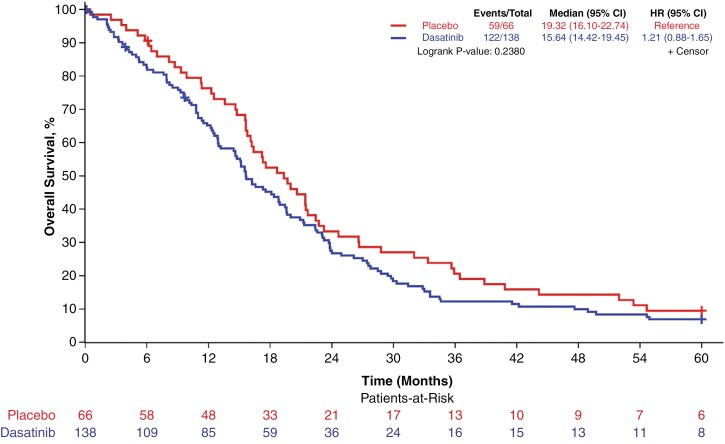
Overall survival according to treatment arm. Overall survival for dasatinib (blue) versus placebo (red), demonstrating no significant differences. HR, hazard ratio.

Of the 204 patients included in the primary analysis, 20 remained alive and not lost to follow-up with a median follow-up of 70.0 months at the time of this analysis. OS at 12, 15, and 18 months for the placebo arm was 76% (95% CI: 67%–88%), 68% (58%–81%), and 52% (41%–66%), respectively. OS at 12, 15, and 18 months for the dasatinib arm was 65% (57%–74%), 55% (47%–64%), and 45% (37%–55%). OS was not significantly different between treatment arms; median OS for the dasatinib arm was 15.6 months compared to 19.3 months for placebo (Figure 2, unadjusted HR for death: 1.21, favoring placebo, 95% CI: 0.88–1.65, log-rank *P*-value: .238). Accordingly, this study did not meet the primary endpoint and we cannot recommend further testing of dasatinib in a phase III trial.

#### Progression-free survival and time-to-progression.—

**Figure 3.**
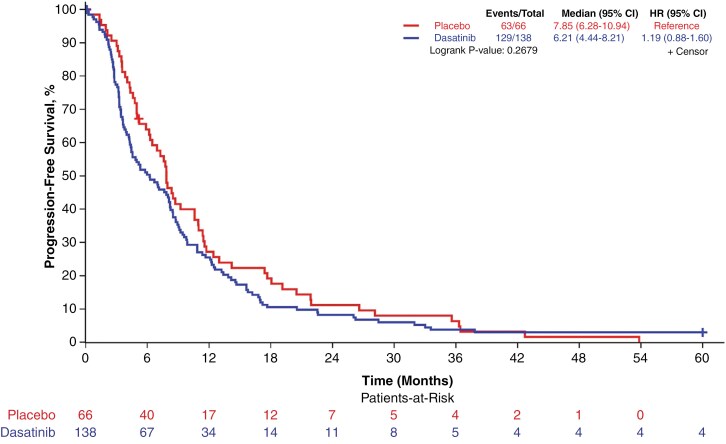
Progression-free survival according to treatment arm. Progression-free survival for dasatinib (blue) versus placebo (red), demonstrating no significant differences. HR, hazard ratio

PFS was not significantly different between treatment arms; median PFS for the dasatinib arm was 6.2 months (95% CI = 4.4–8.2) compared to 7.85 months for the placebo arm (95% CI = 6.28–10.94; log-rank *P*-value: .268; Figure 3). Similarly, there was no significant difference in TTP; the median TPP on the dasatinib arm was 6.7 months (95% CI = 4.6–8.4) and 7.9 months for the placebo arm (95% CI = 6.3–10.93; log-rank *P*-value: .338).

#### MGMT methylation analysis.—

There was no significant difference in OS based on MGMT methylation status between the dasatinib and placebo arms. Patients with MGMT-methylated tumors did not have significantly improved OS with dasatinib compared to placebo (median OS 20.9 months for dasatinib versus 20.0 months for placebo, *P* = .4). Median OS was 20.0 months for patients with MGMT-methylated tumors compared to 15.7 for patients with MGMT-unmethylated tumors (*P* = .27). Similarly, there was no significant difference in PFS between treatment arms based on MGMT status (7.1 months for methylated vs. 5.7 months for unmethylated, *P* = .23). Among patients receiving dasatinib, patients with MGMT-methylated tumors had a statistically significant longer PFS (7.7 months) than those with unmethylated tumors (4.3 months; *P* = .039). There was no difference in PFS or TTP between methylated or unmethylated patients on the placebo arm (*P* = .96 for both).

Multivariable Cox models were used to test for significant interaction between Dasatinib and MGMT methylation by including arm, MGMT status, and an interaction term as independent variables in the models. No statistically significant interactions were observed in any of the models for OS (interaction *P* = .62), PFS (interaction *P* = .18), or TTP (interaction *P* = .19).

#### Adverse events.—

AE data are available for 196 evaluable patients (dasatinib arm = 133, placebo arm = 63). There were significant differences between treatment arms in rates of anemia, lymphopenia, nausea, and increased creatinine (Table 2). There was a significantly higher rate of grade 3 lymphopenia in the placebo arm compared to the dasatinib arm. There were no treatment-related grade 5 AEs reported.

**Table 2.** Rate of Adverse Event by Treatment Arm

Adverse event	Dasatinib (*n* = 133)	Placebo (*N* = 63)	*P*-value*
Hemoglobin decreased			.003
Grade 1	68 (51.1%)	35 (55.6%)	
Grade 2	31 (23.3%)	4 (6.3%)	
Grade 3	9 (6.8%)	1 (1.6%)	
Grade 4	1 (0.8%)	0 (0%)	
Lymphocyte count decreased			.008
Grade 1	14 (10.5%)	4 (6.3%)	
Grade 2	36 (27.1%)	9 (14.3%)	
Grade 3	27 (20.3%)	30 (47.6%)	
Grade 4	10 (7.5%)	5 (7.9%)	
Nausea			.046
Grade 1	48 (36.1%)	28 (44.4%)	
Grade 2	32 (24.1%)	12 (19.0)	
Grade 3	12 (9.0%)	1 (1.6%)	
Creatinine increased			.016
Grade 1	21 (15.8%)	16 (25.4%)	
Grade 2	6 (4.5%)	0 (0%)	
Grade 3	3 (2.2%)	0 (0%)	

*Kruskal–Wallis rank sum test.

#### Quality-of-life results.—

QOL data were available for 196 patients (133 dasatinib arm, 63 on placebo), with 99% of quality-of-life data present at baseline. Patient demographics and baseline QOL scores were well-balanced between arms. At baseline, patients on the dasatinib arm reported more insomnia on the EORTC QLQ-C15 (*P* = .047). No other measures on the FACT BR or EORTC QLQ were significantly different between arms.

Follow-up QOL measurements were conducted at cycles 4, 6, 8, 10, and 12 and were adjusted for age, sex, and corticosteroid therapy. The rate of quality-of-life data captured decreased at later timepoints (cycle 4 = 61%, cycle 6 = 38%, cycle 8 = 25%, cycle 10 = 19%, and cycle 12 = 15%). While there were no significant differences between arms for any follow-up QOL measures, there were significant changes in several domains over time for the patients who did complete surveys (Supplementary Tables 2 and 3). Only the EORTC QLQ-BN20 measure of “itchy skin” was significantly different between treatment arms on mixed models analysis, with patients on the dasatinib arm reporting more itchy skin (*P* = .009).

## Discussion

This multi-institutional randomized clinical trial demonstrated no improvement in progression-free or overall survival with the addition of dasatinib to standard chemoradiation for glioblastoma. While in the future, genomic subsets of glioblastoma that could benefit may be identified, at this time, dasatinib does not appear to be effective in this setting.^11^ A careful introspection and investigation into how and why this drug showed preclinical promise, but ultimately was not successful in patients, may offer perspective for future attempts at improving outcomes for patients with glioblastoma.

Preclinical studies of dasatinib demonstrated the potential to improve outcomes for glioblastoma by overcoming angiogenesis, tumor invasion, and radioresistance.^3,4,6,12–14^ In one study, SFK inhibitors inhibit the invasion of human glioma-implanted spheroids in 3D collagen type I matrices, providing preclinical evidence for a role of Src inhibitors to reduce the invasive potential of malignant gliomas.^3^ Another study demonstrated the synergistic cell-killing effect of dasatinib with temozolomide in melanoma cell lines.^12^ The glioblastoma patient-derived xenograft GBM39 has high-level Src activation, suggesting that SFKs are critical signaling intermediates in at least a subset of GBM, and supporting the hypothesis that dasatinib may improve outcomes when integrated with standard chemoradiation for newly diagnosed glioblastoma.^15^

Beyond the preclinical promise, dasatinib has demonstrated clinical success in other cancers. Dasatinib is approved by the U.S. Food and Drug Administration for the treatment of chronic myelogenous leukemia and has demonstrated promising clinical activity in hormone-refractory prostate cancer, multiple myeloma, gastrointestinal tumors, and advanced solid tumors.^16–20^ Unfortunately, this promising preclinical data and clinical data from other cancers did not translate into improved glioblastoma response in patients enrolling on this clinical trial. Consistent with this study, 2 prospective cooperative group clinical trials evaluating dasatinib for recurrent glioblastoma published after completion of the clinical trial described in this manuscript also did not detect any benefit to the drug.^7,8,21^

These negative results support the axiom that randomized clinical trials remain necessary to confirm or refute the benefit of candidate treatments, regardless of their preclinical promise. Particularly in glioblastoma, candidate therapeutics with preclinical promise have often failed to translate to clinical benefit for patients.^22^ These clinical trials require significant investment of resources and patient commitment, underscoring the need for methods to identify the candidate therapeutics that are most likely to succeed. Setting a high bar for preclinical evidence of candidate therapeutics for glioblastoma prior to testing on large clinical trials in humans has the potential to decrease the rate of future negative studies in this disease.

One potential explanation for the failure of dasatinib and other drugs is the inability to adequately reach tumor cells, despite the long-held and potentially incorrect belief that the blood-brain barrier has been disrupted in glioblastoma.^23,24^ There is increasing clinical evidence that addressing non-contrast enhancing glioblastoma may improve outcomes for patients.^25–28^ Human studies that ensure adequate doses of the drug are reaching higher proportions of brain tumor cells, particularly non-enhancing biologically aggressive tumors, may be indicated prior to large prospective efficacy studies. One way this may be achieved is by giving a candidate drug prior to planned resection, then assessing the concentration in tumor tissue.^29,30^

Despite prior failures, recent success with targeted therapies in IDH-mutant glioma as well as brain metastases provides hope that targeted agents may eventually make clinical headway for glioblastoma.^31,32^ Along these lines, improved molecular subclassification of glioblastoma may identify subgroups of patients most likely to benefit from a given intervention. While no subgroup appeared to benefit from dasatinib on this analysis, a weakness of this clinical trial is the lack of detailed genetic and molecular data that would allow for more thorough biologic subgrouping and potential identification of subgroups who may benefit from dasatinib.

Dasatinib appeared to be relatively well tolerated, though patients had modest increases in hematologic and non-hematologic toxicities. During the early timepoints where more QOL data were available, there did not appear to be significant differences between arms. At later time points, changes in QOL attributable to dasatinib are difficult to assess; there was a substantial amount of missing QOL data, with less than 40% of patients completing surveys at cycle 6 and beyond. Prior statistical reports have found that definitive conclusions cannot be drawn if over 50% of the data are missing [11]. While generalizable conclusions about QOL between arms are not possible from this sample, these results highlight the importance of developing new methodologies to ensure more complete QOL data collection in future trials. Efforts to improve QOL instrument design and collection are underway, with future tools likely to allow for digital, remote collection.

One unexpected toxicity finding was decreased lymphopenia in the dasatinib arm compared to placebo, indicating that the lympho-depleting effects of TMZ may have been modulated by dasatinib. Whether the anti-tumor effects of TMZ could also be modulated by dasatinib is unknown; it is noteworthy that the PFS and OS of the dasatinib arm were numerically worse than placebo, though not statistically significantly different. There are other examples of glioblastoma clinical trials in which the experimental agent resulted in numerically or statistically worse outcomes than standard treatment, further supporting temozolomide and radiation as the standard of care over therapies that have not been proven in randomized controlled trials.^33–35^

In conclusion, this randomized trial did not demonstrate any improvement in progression-free or overall survival with the addition of dasatinib to standard chemoradiation for glioblastoma. New models are needed to generate and investigate preclinical data in glioblastoma to identify the best candidates for prospective trials in human patients.

## Supplementary material

Supplementary material is available online at *Neuro-Oncology* (https://academic.oup.com/neuro-oncology).

noaf156_Supplementary_Tables_S1-S3

## Data Availability

Data is available upon request per Alliance procedures.

## The following institutional networks participated in this study

Altru Cancer Center, Grand Forks, ND; Grant Richard Seeger; Ascension Via Christi Hospitals Wichita, Wichita, KS; Dennis F. Moore; Avera Cancer Institute, Sioux Falls, SD; Bixby Medical Center, Adrian, MI; Cancer Research for the Ozarks NCORP, Springfield, MO; Jay W. Carlson; UG1CA189822; Cancer Research of Wisconsin and Northern Michigan Consortium, Green Bay, WI; Anthony John Jaslowski; Carle Cancer Center NCI Community Oncology Research Program, Urbana, IL; Kendrith M. Rowland; UG1CA189861; Coborn Cancer Center at Saint Cloud Hospital, Saint Cloud, MN; Donald James Jurgens; Columbus NCI Community Oncology Research Program, Columbus, OH; Timothy David Moore; UG1CA189954; CommonSpirit Health Research Institute, Englewood, CO; Shahzad Siddique; Dayton CCOP; Dayton NCI Community Oncology Research Program, Dayton, OH; Howard M. Gross; UG1CA189957; Essentia Health NCI Community Oncology Research Program, Duluth, MN; Bret E.B. Friday; UG1CA189812; Franciscan Health Indianapolis, Indianapolis, IN; Michael Thomas Slaughter; Hartford Hospital, Hartford, CT; Heartland Cancer Research NCORP, Decatur, IL; Bryan A. Faller; UG1CA189830; Hematology Oncology Associates of Central New York-East Syracuse, East Syracuse, NY; Steven M. Duffy; Iowa-Wide Oncology Research Coalition NCORP, Des Moines, IA; Joshua Lukenbill; UG1CA189816; John B Amos Cancer Center, Columbus, GA; Andrew W. Pippas; Mayo Clinic LAPS, Rochester, MN; Steven R. Alberts; UG1CA232760; Memorial Regional Hospital/Joe DiMaggio Children’s Hospital, Hollywood, FL; Luis E. Raez; Metro Minnesota Community Oncology Research Consortium, Saint Louis Park, MN; Daniel M. Anderson; UG1CA189863; Miami Valley Hospital, Dayton, OH; Tarek M Sabagh; Michigan Cancer Research Consortium NCORP, Ann Arbor, MI; Tareq Al baghdadi; UG1CA189971; Mission Hospital, Asheville, NC; Cameron Blair Harkness; Montana Cancer Consortium CCOP; Montana Cancer Consortium NCORP, Billings, MT; John M. Schallenkamp; UG1CA189872; Mount Sinai Hospital, New York, NY; Lewis R. Silverman; NCORP of the Carolinas (Prisma Health NCORP), Greenville, SC; Ki Young Chung; UG1CA189972; New Hampshire Oncology Hematology PA-Concord, Concord, NH; Douglas J. Weckstein; New Mexico Minority Underserved NCORP, Albuquerque, NM; Zoneddy Ruiz Dayao; UG1CA189856; Northern Indiana Cancer Research Consortium, South Bend, IN; ProMedica Flower Hospital, Sylvania, OH; Saint Charles Hospital, Oregon, OH; Sanford NCI Community Oncology Research Program of the North Central Plains, Sioux Falls, SD; Samuel Jacob Milanovich; UG1CA189825; Southeast Cancer Control Consortium CCOP; Toledo Clinic Cancer Centers-Toledo, Toledo, OH; Rex B. Mowat; University of Iowa/Holden Comprehensive Cancer Center, Iowa City, IA; Chandrikha Chandrasekharan; Wake Forest University Health Sciences, Winston-Salem, NC; Heidi Diana Klepin; Washington University - Siteman Cancer Center LAPS, Saint Louis, MO; Nancy L. Bartlett; UG1CA233339; and Wisconsin NCI Community Oncology Research Program, Marshfield, WI; Matthias Weiss; UG1CA189956.
