# Supplementary material for: Final report on North Central Cancer Treatment Group N0877 (alliance): A phase II randomized, placebo-controlled trial of chemoradiotherapy with or without dasatinib for glioblastoma
Source: Neuro Oncol. 2025 Jun 28;27(10):2661–70. doi: 10.1093/neuonc/noaf156 (PMC12833535; doi:10.1093/neuonc/noaf156)
Supplement: noaf156_Supplementary_Tables_S1-S3 [file noaf156_supplementary_tables_s1-s3.docx]

Supplemental Table 1: Phase II Study Treatment

| **Pretreatment medication**  Prophylaxis for Pneumocystis carinii pneumonia ***is required***  (See Sections 7.23 through 7.234). | | | | | |
| --- | --- | --- | --- | --- | --- |
| **Cycle #** | **Agent** | **Dose** | **Route** | **Frequency** | **Cycle length** |
| **Cycle 1**  **Concomitant Chemotherapy**  (RT + TMZ + Dasatinib **or** placebo) | Dasatinib **or** placebo | 150 mg q AM | Oral | Once daily;  Begin on the same day as RT | 42 days^6^ |
|  | Radiation^3^ | 6000 cGy  (200 cGy x  30 fractions) |  | 5 days/week |  |
|  | TMZ^4, 5^ | 75 mg/m^2^ daily | Oral | Daily;  Begin on the same day as RT |  |
| **Cycle 2**  **Rest Period** | **28 to 42 day long rest period^9^** | | | | |
| **Cycle 3**  **Adjuvant**  **Chemotherapy**  (TMZ + Dasatinib **or** placebo) | Dasatinib **or**  placebo^1, 2, 7^ | Continue  at same dose  as end of cycle 1 | Oral | Once daily | 28 days |
|  | TMZ^4^ | 150 mg/m^2^ day | Oral | Daily days 1-5  **only** |  |
| **Cycles 4-8**  **Adjuvant**  **Chemotherapy**  (TMZ + Dasatinib **or** placebo) | Dasatinib **or**  placebo^1, 2, 7^ | Continue  at same dose  as end of cycle 3 | Oral | Once daily | 28 days |
|  | TMZ^4,8^ | 200 mg/m^2^ day | Oral | Daily days 1-5  **only** |  |
| **All**  **subsequent cycles**  **Adjuvant**  **Chemotherapy**    (Dasatinib alone **or** placebo alone) | Dasatinib **or** placebo^1, 2, 7^ | Continue with same dasatinib dose as end of previous cycle | Oral | Once daily | 28 days |

Supplemental Tables 2 and 3: Overall p-values for mixed models adjusting for age, sex, and corticosteroid therapy. Repeated Measures Mixed Models: For each of the quality-of-life (QOL) endpoints, repeated measures models were created using information at baseline and cycles 4, 6, 8, 10, and 12. Models were adjusted for age, sex, and corticosteroid therapy. While there were no significant arm effects for any of the QOL measures, there were significant changes in QOL over time. Itchy skin also had a significant interaction between arm and cycle. Statistically significant findings are highlighted for ease of review.

| **QOL Endpoint** | **Arm** | **Time** | **Interaction** |
| --- | --- | --- | --- |
| MMSE | 0.832 | 0.235 | 0.383 |
| FACT BR Physical Well Being | 0.581 | **<.001** | 0.925 |
| FACT BR Social/Family Well Being | 0.349 | **0.009** | 0.418 |
| FACT BR Emotional Well Being | 0.145 | 0.136 | 0.414 |
| FACT BR Functional Well Being | 0.287 | 0.190 | 0.374 |
| FACT BR FACT-G Score | 0.359 | **<.001** | 0.936 |
| FACT BR Additional Concerns Score | 0.721 | **0.031** | 0.933 |
| FACT BR FACT-BR Total Score | 0.704 | **<.001** | 0.954 |
| EORTC QLQ-C15 Physical Function | 0.544 | **0.003** | 0.942 |
| EORTC QLQ-C15 Emotional Function | 0.142 | 0.545 | 0.384 |
| EORTC QLQ-C15 Overall QOL | 0.261 | 0.052 | 0.706 |
| EORTC QLQ-C15 Fatigue | 0.984 | **0.003** | 0.939 |
| EORTC QLQ-C15 Nausea/Vomiting | 0.821 | **0.001** | 0.172 |
| EORTC QLQ-C15 Pain | 0.240 | 0.349 | 0.557 |
| EORTC QLQ-C15 Dyspnea | 0.262 | 0.178 | 0.082 |
| EORTC QLQ-C15 Insomnia | 0.060 | **<.001** | 0.280 |
| EORTC QLQ-C15 Appetite Loss | 0.751 | **<.001** | 0.210 |
| EORTC QLQ-C15 Constipation | 0.956 | 0.305 | 0.220 |
| EORTC QLQ-BN20 Future Uncertainty | 0.538 | 0.064 | 0.400 |
| EORTC QLQ-BN20 Visual Disorder | 0.091 | 0.996 | 0.520 |
| EORTC QLQ-BN20 Motor Disorder | 0.831 | 0.131 | 0.613 |
| EORTC QLQ-BN20 Communication Deficit | 0.903 | 0.076 | 0.830 |
| EORTC QLQ-BN20 Headaches | 0.787 | 0.353 | 0.841 |
| EORTC QLQ-BN20 Seizures | 0.434 | 0.317 | 0.682 |
| EORTC QLQ-BN20 Drowsiness | 0.128 | **0.012** | 0.480 |
| EORTC QLQ-BN20 Hair Loss | 0.944 | **<.001** | 0.883 |
| EORTC QLQ-BN20 Itchy Skin | 0.378 | **0.001** | **0.009** |
| EORTC QLQ-BN20 Weakness of Legs | 0.394 | **0.026** | 0.460 |
| EORTC QLQ-BN20 Bladder Control | 0.695 | 0.437 | 0.879 |

|  |  |  |  |  |  |  | **Interactions** | | | | |
| --- | --- | --- | --- | --- | --- | --- | --- | --- | --- | --- | --- |
| **QOL Endpoint** | **Dasatinib** | **Cycle 4** | **Cycle 6** | **Cycle 8** | **Cycle 10** | **Cycle 12** | **Cycle 4*Dasat** | **Cycle 6*Dasat** | **Cycle 8*Dasat** | **Cycle 10*Dasat** | **Cycle 12*Dasat** |
| MMSE | 0.832 | 0.097 | 0.170 | 0.778 | 0.759 | 0.999 | 0.609 | 0.078 | 0.772 | 0.907 | 0.683 |
| FACT BR Physical Well Being | 0.581 | **<.001** | **0.007** | **0.008** | 0.604 | 0.327 | 0.689 | 0.930 | 0.586 | 0.558 | 0.502 |
| FACT BR Social/Family Well Being | 0.349 | **0.003** | **0.007** | **0.011** | 0.277 | 0.222 | 0.423 | 0.126 | **0.048** | 0.738 | 0.703 |
| FACT BR Emotional Well Being | 0.145 | 0.617 | 0.213 | 0.719 | 0.717 | 0.416 | 0.173 | 0.948 | 0.435 | 0.385 | 0.379 |
| FACT BR Functional Well Being | 0.287 | 0.707 | 0.433 | 0.623 | 0.185 | 0.544 | 0.120 | 0.261 | 0.229 | 0.069 | 0.959 |
| FACT BR FACT-G Score | 0.359 | **0.003** | **0.038** | 0.099 | 0.823 | 0.724 | 0.375 | 0.874 | 0.885 | 0.912 | 0.873 |
| FACT BR Additional Concerns Score | 0.721 | 0.188 | 0.083 | 0.132 | 0.232 | 0.415 | 0.408 | 0.779 | 0.837 | 0.756 | 0.836 |
| FACT BR FACT-BR Total Score | 0.704 | **0.005** | **0.018** | **0.049** | 0.382 | 0.581 | 0.459 | 0.901 | 0.760 | 0.868 | 0.889 |
| EORTC QLQ-C15 Physical Function | 0.544 | **0.008** | 0.084 | 0.070 | 0.506 | 0.537 | 0.548 | 0.906 | 0.612 | 0.783 | 0.745 |
| EORTC QLQ-C15 Emotional Function | 0.142 | 0.983 | 0.125 | 0.587 | 0.742 | 0.331 | 0.237 | 0.306 | 0.491 | 0.523 | 0.368 |
| EORTC QLQ-C15 Overall QOL | 0.261 | 0.702 | 0.234 | 0.633 | 0.492 | 0.360 | 0.093 | 0.377 | 0.612 | 0.680 | 0.685 |
| EORTC QLQ-C15 Fatigue | 0.984 | **0.011** | 0.262 | 0.364 | 0.880 | 0.688 | 0.923 | 0.421 | 0.936 | 0.790 | 0.679 |
| EORTC QLQ-C15 Nausea/Vomiting | 0.821 | **0.018** | 0.893 | 0.954 | 0.820 | 0.655 | 0.412 | **0.015** | 0.475 | 0.904 | 0.575 |
| EORTC QLQ-C15 Pain | 0.240 | 0.365 | 0.389 | 0.951 | 0.630 | 0.285 | 0.921 | 0.101 | 0.348 | 0.781 | 0.694 |
| EORTC QLQ-C15 Dyspnea | 0.262 | 0.674 | 0.455 | 0.114 | 0.724 | 0.361 | **0.040** | **0.009** | 0.975 | 0.403 | 0.262 |
| EORTC QLQ-C15 Insomnia | 0.060 | 0.266 | 0.378 | 0.624 | 0.268 | 0.138 | 0.126 | **0.048** | **0.048** | 0.240 | 0.279 |
| EORTC QLQ-C15 Appetite Loss | 0.751 | **<.001** | **<.001** | **0.048** | 0.707 | 0.602 | 0.144 | **0.026** | 0.942 | 0.790 | 0.720 |
| EORTC QLQ-C15 Constipation | 0.956 | 0.429 | **0.033** | 0.194 | 0.366 | 0.156 | 0.563 | 0.199 | 0.625 | 0.194 | **0.015** |
| EORTC QLQ-BN20 Future Uncertainty | 0.538 | 0.891 | 0.802 | 0.290 | 0.273 | 0.283 | 0.763 | **0.038** | 0.973 | 0.594 | 0.476 |
| EORTC QLQ-BN20 Visual Disorder | 0.091 | 0.621 | 0.495 | 0.966 | 0.493 | 0.483 | 0.369 | 0.230 | 0.790 | 0.190 | 0.102 |
| EORTC QLQ-BN20 Motor Disorder | 0.831 | 0.369 | 0.644 | 0.999 | 0.826 | 0.816 | 0.244 | 0.107 | 0.561 | 0.665 | 0.853 |
| EORTC QLQ-BN20 Communication Deficit | 0.903 | 0.579 | 0.143 | 0.824 | 0.653 | 0.594 | 0.995 | 0.835 | 0.582 | 0.838 | 0.217 |
| EORTC QLQ-BN20 Headaches | 0.787 | 0.289 | 0.923 | 0.556 | 0.213 | 0.892 | 0.8418 | 0.840 | 0.396 | 0.476 | 0.607 |
| EORTC QLQ-BN20 Seizures | 0.434 | 0.688 | 0.824 | 0.238 | 0.521 | 0.592 | 0.290 | 0.259 | 0.640 | 0.583 | 0.838 |
| EORTC QLQ-BN20 Drowsiness | 0.128 | **0.015** | 0.243 | **0.012** | 0.069 | 0.341 | 0.710 | 0.983 | 0.198 | 0.127 | 0.203 |
| EORTC QLQ-BN20 Hair Loss | 0.944 | **<.001** | **0.046** | 0.424 | 0.582 | 0.359 | 0.844 | 0.957 | 0.771 | 0.411 | 0.464 |
| EORTC QLQ-BN20 Itchy Skin | 0.378 | 0.119 | 0.848 | **0.022** | **0.005** | 0.060 | 0.375 | **0.038** | 0.701 | 0.081 | 0.053 |
| EORTC QLQ-BN20 Weakness of Legs | 0.394 | 0.135 | 0.441 | 0.147 | 0.964 | 0.595 | 0.5291 | 0.170 | 0.673 | 0.482 | 0.411 |
| EORTC QLQ-BN20 Bladder Control | 0.695 | 0.187 | 0.794 | 0.168 | 0.856 | 0.669 | 0.682 | 0.945 | 0.641 | 0.406 | 0.604 |
